# Vitamin B12 Deficiency in Relation to Functional Disabilities

**DOI:** 10.3390/nu5114462

**Published:** 2013-11-12

**Authors:** Breanna S. Oberlin, Christy C. Tangney, Kristin A. R. Gustashaw, Heather E. Rasmussen

**Affiliations:** 1 Diabetes Research Group, Seattle Institute of Biomedical and Clinical Research, 1660 S. Columbian Way, DPP-151, Seattle, WA 98108, USA; E-Mail: breanna.oberlin@gmail.com; 2 Department of Clinical Nutrition, Rush University Medical Center, 1700 West Van Buren St., Suite 425, Chicago, IL 60612, USA; E-Mails: kristin_a_gustashaw@rush.edu (K.A.R.G.); heather_rasmussen@rush.edu (H.E.R.)

**Keywords:** vitamin B12 deficiency, methylmalonic acid, homocysteine, peripheral neuropathy, functional impairment

## Abstract

This study was designed to assess whether symptoms, functional measures, and reported disabilities were associated with vitamin B12 (B12) deficiency when defined in three ways. Participants, aged 60 or more years of age, in 1999–2002 National Health and Nutrition Examination Surveys (NHANES) were categorized in relation to three previously used definitions of B12 deficiency: (1) serum B12 < 148 pmol/L; (2) serum B12 < 200 pmol/L and serum homocysteine > 20 μmol/L; and (3) serum B12 < 258 pmol/L or serum methylmalonic acid > 0.21 μmol/L. Functional measures of peripheral neuropathy, balance, cognitive function, gait speed, along with self-reported disability (including activities of daily living) were examined with standardized instruments by trained NHANES interviewers and technicians. Individuals identified as B12 deficient by definition 2 were more likely to manifest peripheral neuropathy OR (odds) (95% confidence intervals), *p* value: 9.70 (2.24, 42.07), 0.004 and report greater total disability, 19.61 (6.22, 61.86) 0.0001 after adjustments for age, sex, race, serum creatinine, and ferritin concentrations, smoking, diabetes, and peripheral artery disease. Smaller, but significantly increased, odds of peripheral neuropathy and total disability were also observed when definition 3 was applied. Functional measures and reported disabilities were associated with B12 deficiency definitions that include B12 biomarkers (homocysteine or methylmalonic acid). Further study of these definitions is needed to alert clinicians of possible subclinical B12 deficiency because functional decline amongst older adults may be correctable if the individual is B12 replete.

## 1. Introduction

Physical disability and cognitive impairment are disorders common among older adults and represent a major public health concern. Older adults are also at greater risk of vitamin B12 (B12) deficiency, which is characterized by sensory disturbances and neurological abnormalities, including cognitive decline, peripheral neuropathy, decreased muscle strength, and functional disability [1,2]. As there appears to be no one indicator that, alone, ascertains whether an individual is either becoming depleted or deficient in B12 [1], several costly and time-consuming biomarkers are used to assess B12 status. Serum B12 concentrations alone are not necessarily the best indicator for low B12 nutriture since these may be maintained at the expense of tissue depletion [1,3]. Thus, it is important to confirm deficiency using additional measurements of biochemical or clinical indicators of B12 nutriture [3].

B12 is a cofactor for methylation reactions involving methylmalonic acid (MMA) and total homocysteine (homocysteine). Both are elevated when concentrations of B12 are inadequate [1,2,3]. Some researchers use homocysteine [2,4,5,6,7] as an indicator of B12 nutriture, yet homocysteine is not only an indicator of B12 deficiency; deficiencies of vitamin B6 and folate can result in a buildup in homocysteine concentrations. The definition of B12 deficiency chosen that incorporates B12 and homocysteine values has been used to describe the prevalence of “metabolically significant” deficiency in three different British national cohorts of older adults [2]. Morris *et al.* [8] used serum concentrations of B12 or MMA (B12 < 148 pmol/L or MMA > 0.21 μmol/L) to characterize deficiency, while Johnson and coworkers [9], Pennypacker and colleagues [10], and Stabler and coworkers [11] have used a more liberal cutoff for B12 (<258 pmol/L) and elevated MMA (>0.27 μmol/L). Several groups have found that B12 concentrations as high as 258 pmol/L are accompanied by the presence of abnormal physical signs and symptoms (impaired reflexes, impaired cognitive function, and missing ankle tendon jerks) consistent with B12 deficiency [12]: however, many of these findings are based either on case series reports or in cohorts outside the US where food fortification policies are different. While there is general consensus that individuals with levels exceeding the common B12 cutoff (<148 pmol/L) may develop neurologic abnormalities and other signs and symptoms, to the best of our knowledge, there is no national probability-based survey of these symptoms in the context of definitions of vitamin B12 nutriture that use homocysteine or MMA with B12 available in the literature. We were particularly interested in applying a definition with a higher B12 cutoff (<258 pmol/L) with the specific B12 marker MMA to assess whether physical and sensory impairments might be detected.

The objectives of this study were to describe (1) the prevalence of B12 deficiency in older adults on the basis of three definitions; first, the traditional definition using serum B12 alone (<148 pmol/L); second, serum B12 (<200 pmol/L) and homocysteine (>20 μmol/L); and third, serum B12 < 258 pmol/L or MMA > 0.21 μmol/L, and (2) the relations of these B12 deficiency definitions to functional measures available in the National Health and Nutrition Examination Survey (NHANES) for 1999–2000 and 2001–2002. We selected functional measures that historically have been described for B12 deficiency to determine whether the symptoms and functional measures/disabilities common to this age group and characteristic of B12 deficiency were observed in participants meeting any of the proposed B12 definitions.

## 2. Methods

### 2.1. Study Design

We examined all adults 60 years and older in NHANES 1999–2000 and 2001–2002 (NHANES 1999–2002). We used these data sets as several functional measures and markers of B12 status were not available in NHANES 2003–2004, 2005–2006, or in more recent surveys (e.g., balance, lower extremity function, peripheral neuropathy, cognitive function examination, and serum MMA concentrations). This report is based on the 3105 persons aged 60 years or more who participated in these surveys and had values for serum vitamin B12, homocysteine, and MMA.

### 2.2. Biochemical Measurements

In NHANES, the Quantaphase II Folate/Vitamin B12 radioassay kit (Bio-Rad Laboratories, Hercules, CA, USA) was used to measure serum B12 and folate concentrations. Plasma aliquots were analyzed for MMA by gas chromatography-mass spectrometry using the reference range for MMA of 0.06 to 0.21 μmol/L for adults with normal creatinine concentrations [13]. Serum creatinine levels were measured using a Jaffe rate reaction; renal insufficiency was defined at levels >120 μmol/L for women and >133 μmol/L for men [8]. Plasma homocysteine and mean cell volume are additional measures of B12 nutriture. Plasma homocysteine concentrations were measured using a fully automated fluorescence polarization immunoassay (Abbott Diagnostics, Abbott Park, IL, USA). Mean cell volumes greater than or equal to 99 fL were considered indicative of macrocytosis [8]. Serum ferritin was acquired to rule out possible confounding affect of inadequate levels. Serum ferritin levels were measured using the QuantImune Ferritin IRMA kit (Bio-Rad Laboratories, Hercules, CA, USA). B12 deficiency was defined as follows: (1) B12 < 148 pmol/L (traditional cutoff); (2) B12 < 200 pmol/L and homocysteine > 20 μmol/L; and (3) B12 < 258 pmol/L or MMA > 0.21 μmol/L.

### 2.3. Functional Measures

#### 2.3.1. Peripheral Neuropathy Assessment

Two sources in NHANES 1999–2002 were available to assess peripheral neuropathy: (1) a self administered physical functioning questionnaire and also another one for diabetes in which subjects self-reported the presence or absence of physical function impairments or specific symptoms of all adults 40 years and older, and (2) the lower extremity disease examination of insensate areas on both feet through monofilament testing. For self-reported peripheral neuropathy, the respondent had to answer affirmatively to two questions about numbness in hands or feet in past 3 months (other than from falling asleep) and where this numbness was experienced. Another pair of questions address whether the respondent experienced a painful sensation or tingling in hands or feet in the past 3 months, and then at which sites this had occurred. For the second, the examination component, monofilament testing was conducted measuring three points on each foot: the bottom of the foot at the first metatarsal head, fifth metatarsal head, and halux. An area was insensate if, in two out of three trials, the subject incorrectly identified when the instrument was placed. Peripheral neuropathy was defined by the determination of one or more insensate areas [13,14].

#### 2.3.2. Balance

Balance was assessed through a pre-examination questionnaire (inquiries of medications, problems or procedures that resulted in balance impairment) and balance testing. Balance testing was scored on a pass/fail basis. A modified Romberg test to assess balance (the vestibular system, vision, and proprioception) was used with four progressively more difficult test conditions; each condition was performed twice. According to the NHANES procedure manuals, only adults aged 60–69 years in the 1999–2000 survey were tested, but in 2001–2002, all subjects above 60 years of age were eligible for testing. For purposes of the current study only, the most difficult test condition (condition 4 testing vestibular function) was used. This procedure tests one’s ability to stand unassisted without the aid of vision or proprioception; subjects stand on a foam-padded surface within their eyes closed. As with previous test conditions, assessment of this outcome was based on the sum of the two possible trials.

#### 2.3.3. Cognitive Functioning

The Digital Symbol Substitution Test (DSST), an executive function measure from the Wechsler Adult Intelligence Scale, third edition [15], was administered during an in-home interview. The maximum score for this test is 133 points, which is reflective of the number of correct symbols drawn within the 120-second time-period. The participant draws symbols to corresponding numbers provided in the key. The test is regarded as a sensitive one for cognitive impairment and captures the respondent’s aptitude in response speed, sustained attention, associative learning, visuospatial skills, and memory. For this study, cognitive impairment was considered to be a score of less than 28, which was the 25th percentile for the distribution of the study sample.

#### 2.3.4. Gait Speed

Gait speed was measured according to standardized procedures and expressed in seconds to complete a 20-foot walk [13,14,16,17]. Habitual gait speed was chosen as a measure of walking function because it may predict subsequent disability [18].

#### 2.3.5. Disability Domains

Participants were also interviewed about functional ability using the physical function questionnaire, an instrument designed to ascertain the levels of dependence in performing tasks. Possible disability was classified into four domains as originally described by Nagi [19], using 12 out of the 19 questions on the instrument; these include: (1) activities of daily living (ADL); (2) instrumental activities of daily living (IADL); (3) leisure and social activities (LSA); and (4) lower extremity mobility (LEM). Examples of the questions for the ADL domain included those about difficulty in eating, getting in and out of bed or dressing yourself. For the IADL domain, such examples include reported difficulties in managing money, doing house chores, or preparing meals. For the LSA, these queries include difficulty in attending social events or going to a movie. For LEM, a respondent might answer affirmatively to difficulty in walking up ten steps or walking for a quarter of a mile. A participant’s answer was coded as affirmative if a person reported “much difficulty” or “unable to do the activity”. Study participants were classified as having one of these disabilities if they reported any of the symptoms queried. A sum of all four domains was also calculated to determine total disability.

#### 2.3.6. Medical Conditions

Presence of diabetes and coronary heart disease was self-reported in the interview. Another condition that may affect lower extremity function, peripheral artery disease (PAD), was assessed in NHANES in the physical function examination. This assessment is one of two components of the lower extremity function examination in NHANES; the second is the peripheral insensate neuropathy testing using monofilament testing defined previously (2.3.1). PAD was assessed by the ankle-brachial index of <0.9 in either leg using an 8.1-MHz Doppler probe (Parks Medical Electronics, Inc., Aloha, Oregon, USA) [13].

#### 2.3.7. Anthropometrics, Smoking, Alcohol, and Supplement Use

Height and weight were measured using standard protocols and calibrated equipment in the mobile examination center. Participants reported if they were a current smoker, the average number of cigarettes smoked per day, and if they drank more than 5 alcoholic drinks a day at any one time. During the household interview, participants were asked about the ingestion of any vitamin and mineral supplements in the past 30 days. A participant was classified as a consumer of supplements containing B12 if he or she reported consuming any supplement with B12 during the past 30 days [13]; no B12 injections were included.

#### 2.3.8. Statistics

The Complex Sample module of SPSS version 17.0 (SPSS Inc., Chicago, IL, USA) was used for statistical analyses; the module allows specification of strata and primary sampling units and applies appropriate sampling weights for study subjects. Data sets from NHANES 1999–2000 and 2001–2002 were combined according to the NHANES analytic guidelines. All outcome variables (biochemical markers, clinical signs, symptoms, and total scores) were assessed for normality by examination of histograms, and transformations were performed if necessary. Natural logarithms of serum ferritin and folate values were used for all subsequent analyses. Significance was assessed with an *a priori* level of <0.05.

Descriptive statistics for the analytic sample of 3105 persons were performed using general linear models for continuous variables with adjustment for the variables included in the basic model: age, sex, race/ethnicity, smoking status, serum creatinine, serum ferritin, and supplement usage. With categorical outcomes such as the proportion of females or non-Hispanic whites, cross tabulations are presented with no adjustments for possible covariates. Geometric means (±standard error or SE) were provided based on these analyses.

In our primary analyses, we estimated the prevalence of B12 deficiency when defined by the previously described definitions using cross-tabulations. These cross-tabulations were repeated for each age group (60–69, 70–79 and 80–89 years). Independence of those associations was assessed using the Rao-Scott chi-square test. In effort to better describe the characteristics or attributes of those adults identified as deficient as compared to those who were not, general linear models were run for continuous variables, and logistic regression for categorical variables. The basic model includes adjustment for the previously described variables and diabetes and peripheral artery disease. Secondary models included B12 supplement use, coronary heart disease, and alcohol use (5 or more drinks per day). Using logistic regression, odds ratios of B12 deficiency were estimated with adults in the non-deficient group serving as the reference group.

## 3. Results

### 3.1. Demographic Characteristics of Study Population

NHANES 1999–2002 participants in this sample averaged [mean (SE)] 68.9 (0.4) years of age and were overweight [BMI = 27.1 (0.4)] but had high-normal serum concentrations of ferritin, folate and MMA, but relatively normal concentrations of B12 and homocysteine (Table 1). Women represented 56.7% of the study participants, and most participants were non-Hispanic white (81.5%). Adults habitually walked the 20-foot test in an average of 7.7 (0.2) seconds or at a speed of 0.79 meters/second. Nearly a quarter of the sample reported current smoking. The average number of cigarettes smoked per day bore a positive relationship with serum cotinine concentrations (β = 0.028, SE = 0.005, *p* = 0.0005) (data not shown). Fourteen percent of the population reported having diabetes. Fifteen percent of the population reported consuming 5 alcoholic drinks per day, which was associated with increased serum γ-glutamyl transferase concentrations (β = 0.0003, SE = 0.001, *p* = 0.017) (data not shown). Based on the cutoff for the DSST, the cognitive function test, 14% of subjects were deemed cognitively impaired. When comparing the groups by the different deficiency criteria, adults classified B12 deficient by the 2nd definition were older with higher proportions of peripheral artery disease, coronary heart disease, diabetes, and had higher creatinine concentrations. Interestingly, the proportion reporting B12 supplement use was higher in this group and similar to that of the total sample. It is important to note that this value reflects reported use of a B12 supplement at least once in the past 30 days, not once every day. The proportion of study participants who reported taking B12-containing supplements differed between race/ethnic groups (data not shown). Nearly 45% of non-Hispanic whites reported taking B12-containing supplements, but only 23% of non-Hispanic blacks and 26% of Mexican-Americans consumed these supplements (data not shown). More females reported taking supplements (44%) than did males (36%). When stratified by gender and race/ethnicity, the proportion of females in each race/ethnic group that took B12 containing supplements was also higher than that of men (data not shown).

**Table 1:** Demographic characteristics of all National Health and Nutrition Examination Surveys (NHANES) 1999–2002 participants 60 years and older in the analytic sample and those classified as vitamin B12 deficient on the basis of three different definitions.

Characteristics	Total Sample (*n* = 3105)	>Deficiency Definitions ^1^		
		B12 Alone (<148 pmol/L) (*n* = 95)	B12 (<200 pmol/L) and Homocysteine (>20 μmol/L) (*n* = 40)	B12 (<258 pmol/L) or MMA (>0.21 μmol/L) (*n* = 1160)
Age (y)	68.9 (0.4) ^2^	70.9 (1.1)	76.6 (1.6)	69.9 (0.5)
Female (%)	56.7 (0.7) ^3^	58.1 (5.3)	78.3 (8.0)	54.7 (1.3)
NonHispanic White (%)	81.5 (2.1)	81.7 (6.2)	87.1 (4.9)	82.8 (2.1)
Smokers ^4^ (%)	23.3 (1.3)	31.4 (8.9)	NR	26.2 (2.1)
Body Mass Index, BMI (kg/m^2^)	27.1 (0.4)	28.9 (1.0)	28.9 (1.3)	27.6 (0.5)
Peripheral artery disease ^5^ (%)	6.2 (0.7)	5.5 (3.0)	24.7 (17.0)	8.9 (1.5)
Coronary heart disease ^6^ (%)	10.3 (0.8)	11.1 (3.9)	30.4 (7.5)	10.3 (0.9)
Diabetes ^7^ (%)	14.0 (1.4)	6.7 (3.8)	19.6 (8.5)	16.7 (1.2)
B12 supplement use ^8^ (%)	41.2 (1.7)	24.9 (7.2)	40.3 (8.1)	29.6 (1.8)
Gait Time ^9^ (in seconds)	7.7 (0.2)	7.2 (0.4)	8.6 (1.0)	7.9 (0.2)
Hematocrit	42.4 (0.2)	42.6 (0.4)	39.6 (0.9)	42.1 (0.2)
Macrocytosis ^10^ (%)	4.7 (0.5)	5.5 (2.1)	7.0 (4.1)	5.6 (0.7)
Creatinine (μmol/L)	81.1 (1.9)	85.8 (4.6)	118.4 (11.4)	94.2 (3.1)
Ferritin (μg/L)	111.0 (10.0)	116.0 (17.3)	95.1 (24.1)	146.9 (7.8)
Folate (nmol/L)	42.9 (1.9)	35.5 (3.0)	40.0 (8.4)	39.8 (1.3)
B12 (pmol/L)	276.6 (12.0)	149.9 (20.7)	179.7 (61.9)	260.5 (8.5)
Homocysteine (μmol/L)	15.1 (2.4)	19.9 (4.8)	37.6 (6.6)	12.4 (0.5)

^1^ No statistical comparisons were conducted; ^2^ Values represent geometric means and standard error (SE) from general linear models with adjustment for the variables included in the basic model; age, sex, race/ethnicity, smoking status, serum creatinine, and serum ferritin, and supplement usage; ^3^ Values represent sample-weighted mean percentages and SE with no adjustments, measured by cross tabulations; ^4^ Smoking status as a current smoker by self-report; NR = no one reported being a current smoker; ^5^ Peripheral artery disease defined as ankle-brachial pressure index (ABI) of <0.9; ^6^ Disease conditions defined by self-report; ^7^ Disease conditions defined by self-report Individuals reporting borderline diabetes categorized as not having diabetes; ^8^ Percent self-reported supplement use of B_12_ containing supplements in the past 30 days; ^9^ Gait time in seconds to complete a 20-foot walk; ^10^ Defined by elevated mean cell volume (MCV) of ≥ 99 fL.

### 3.2. Prevalence of B12 Deficiency

**Figure 1:**
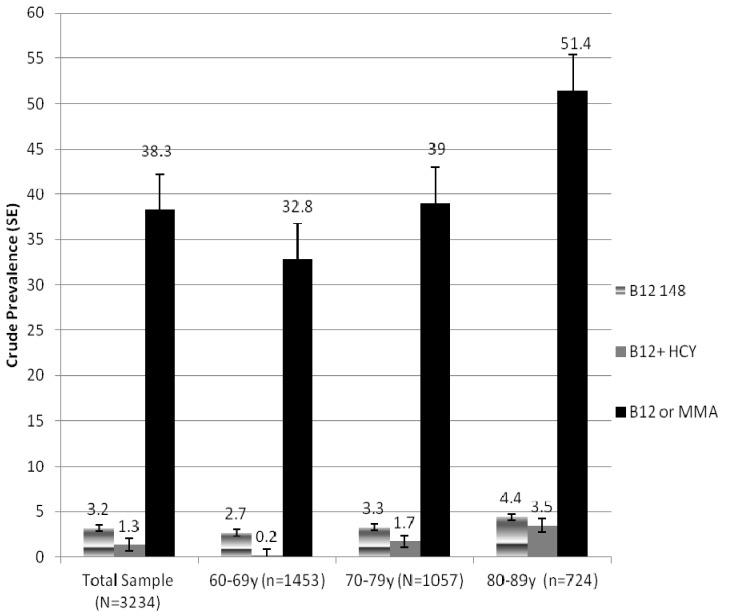
Crude (unadjusted) prevalence of vitamin B12 deficiency for the total population and by age decile based on three definitions of deficiency. Bars from left to right: serum B12 < 148 pmol/L alone (**gradient**); definition 2, serum B12 < 200 pmol/L and homocysteine > 20 μmol/L (**solid gray**) and; the definition 3, serum B12 < 258 pmol/L or MMA > 0.21 μmol/L (**solid black**).

Exactly 3.2% of the sample was deemed B12 deficient based on definition 1, the traditional cutoff of <148 pmol B12/L, 1.3% by definition 2 (serum B12 < 200 pmol/L and serum homocysteine > 20 μmol/L), and 38.3% by definition 3 (serum B12 < 258 pmol/L or serum MMA > 0.21 μmol/L) (Figure 1). An increase in prevalence rates across age deciles is evident (*p* < 0.005).

### 3.3. Functional Measures and Reported Disabilities Associated with B12 Deficiency

None of the functional measures was associated with B12 nutriture on the basis of the traditional cutoff, serum B12 concentrations < 148 pmol/L, except for reported total disability and cognitive performance as measured by DSST score (Table 2). Adults with serum concentrations less than 148 pmol/L were significantly more likely to be cognitively impaired. A similar relation was noted for persons classified deficient on the basis of the definition 3, Odds Ratio = 1.58 (1.01, 2.47), *p* = 0.04. On the basis of definition 2 (B12/homocysteine), the confidence intervals of most measures were very large because of the small number of individuals classified as deficient (*n* = 40). Nevertheless, persons so classified were at markedly greater odds of peripheral neuropathy upon examination [OR = 9.70 (2.24, 42.07), *p* = 0.004] and for reporting total disability and, in particular, lower extremity mobility [OR = 7.58 (1.22, 50.30), *p* = 0.03]. When definition 3 was used, subjects were more likely to have peripheral neuropathy [OR = 1.44 (1.03, 2.02), *p* = 0.03], total disability [OR = 1.60 (1.09, 2.34), *p* = 0.02], and disability in leisure and social activity [1.63 (1.06, 2.51), *p* = 0.03].

**Table 2:** Odds of physical impairments and reported disabilities in 3015 persons 60 years and older from NHANES 1999–2000 and 2001–2002 as a function of three B12 deficiency definitions.

Characteristic	B12 Alone (<148 pmol/L)	B12 (<200 pmol/L) and Homocysteine (>20 μmol/L)	B12 (<258 pmol/L) or MMA (>0.21 μmol/L)
	Odds Ratio; (95% Confidence Intervals); *p* Value		
*Peripheral Neuropathy* ^1^			
Exam % insensate	0.67; (0.25, 1.78); 0.36 ^2^	9.70; (2.24,42.07); 0.004	1.44; (1.03, 2.02); 0.03
*Balance* ^3^			
% fail	2.08; (0.58, 7.44); 0.25	1.82; (0.20, 16.39); 0.58	0.97; (0.57, 1.64); 0.90
*Disability* ^4^			
Total	1.80; (1.03, 3.14); 0.04	19.61; (6.22, 61.86); 0.0001	1.60; (1.09, 2.34); 0.02
Activities of Daily Living (ADL)	0.83; (0.12, 5.77); 0.84	2.20; (0.30, 15.88); 0.42	1.57; (0.78, 3.19); 0.98
Instrumental Activities of Daily Living (IADL)	1.52; (0.46, 5.02); 0.48	2.05; (0.28, 15.20); 0.47	1.36; (0.89, 2.08); 0.15
Lower extremity mobility (LEM)	1.53; (0.65, 3.57); 0.32	7.58; (1.22, 50.30); 0.03	1.44; (0.99, 2.20); 0.06
Leisure and social activities (LSA)	1.35; (0.04, 4.06); 0.58	4.04; (0.38, 42.61); 0.24	1.63; (1.06, 2.51); 0.03
*Cognitive Impairment* ^5^			
Digit Symbol Substitution Test (DSST)	3.62; (1.45, 9.04); 0.01	0.84; (0.03,23.38); 0.91	1.58; (1.01, 2.47); 0.04

^1^ Peripheral Neuropathy is defined as having one or more insensate areas. Insensate areas were determined by monofilament testing completed on three points of each foot; ^2^ Multivariate adjusted odds ratios; 95% confidence intervals; *p* value where the basic model includes age, sex, race/ethnicity, smoking status, serum creatinine, serum log normal ferritin, diabetes, and peripheral artery disease; ^3^ Advanced Romberg: Study participants’ inability to complete or pass the fourth and most difficult balance test of the Romberg Balance Examination; ^4^ Disability is defined as impairment in at least one of the following domains: ADL, IADL, LSA, LEM. Disability within the four domains is defined through the study participant reporting in a self-report questionnaire the inability to complete a task within the given domain; ^5^ Cognitive impairment: ≤28 is less than 25th percentile distribution for the DSST for the sample population, where DSST is the Digit Symbol Substitution Test.

## 4. Discussion

In the present study of a nationally representative sample of older Americans aged 60 years and older, we provide information regarding biochemical definitions of B12 deficiency with respect to signs and symptoms historically associated with B12 deficiency. Several definitions of B12 deficiency were examined because of the concern that subtle signs might be missed if only the most commonly used cutoff was employed (serum B12 concentrations < 148 pmol/L). Because of the potential for irreversible neurological abnormalities associated with B12 deficiency [20,21], it is imperative to identify the biochemical indicators, signs and symptoms that alert the clinician to possible B12 deficiency. Several biochemical definitions have been proposed by researchers to identify B12 deficiency. These include using higher cutoffs for serum B12 in combination with other metabolic markers, such as holotranscobalamin, homocysteine, and MMA [5,7,10,11,12,20,21].

As expected, when the lower, more common cutoff for B12 (<148 pmol/L) was used, prevalence of deficiency was low (3.2%) compared to 38.3% for definition 3. When the B12/homocysteine definition was applied, the lowest prevalence was observed, yet this definition was successful at predicting greater odds of peripheral neuropathy, LEM and total disability. Persons defined by the definition 3 (which reflects a much higher B12 cutoff paired with the specific biomarker, MMA) were also at greater odds of having peripheral neuropathy and LSA disability.

As mentioned previously, several researchers have found signs and symptoms (cognitive impairment, missing ankle tendon jerk, sensory abnormalities, and diminished vibratory sense) in individuals with serum B12 concentrations higher than the more common cutoff of <148 pmol/L [5,7,10,11,12,20,21]. These findings provided us with a rationale to explore different definitions of B12 deficiency wherein physical signs, symptoms, and functional disability may be observed. Based on the results in the present study, total disability and increased odds of cognitive impairment were observed when subjects were classified deficient on the basis of serum B12 concentrations (<148 pmol/L) alone. On the basis of a slightly higher B12 (<200 pmol/L) cutoff and elevated homocysteine concentrations, increased odds of neuropathy, total disability, and lower extremity immobility were observed, though these estimates should be interpreted with caution because of the wide confidence intervals noted. Similarly, the addition of MMA to the higher B12 cutoff (<258 pmol/L) or definition 3 was associated with higher likelihood of peripheral neuropathy in contrast to B12 (<148 pmol/L) definition 1 where such signs were not observed.

Sensory disturbances have also been reported by several researchers in relation to B12 deficiency [5,12,20]. In the present study, we also found increased odds of peripheral neuropathy among individuals defined as B12 deficient by two of the three definitions. Sensory disturbances according to Healton *et al.* [20] were often the first and most common set of symptoms experienced by B12 deficient individuals (more than 70%). In the InCHIANTI cohort, Leishear and coworkers [5] observed that older participants whose homocysteine levels became elevated after a six-year follow-up were at greater odds of being unable to detect monofilament when compared to those with sustained normal homocysteine levels (OR = 5.4, 1.5–19.0); these individuals also exhibited significantly lower nerve conduction velocities in comparison to those with normal homocysteine concentrations. Gregg and coworkers [14] found that the prevalence of peripheral neuropathy rose with increasing age, though they did not examine these symptoms in the context of B12 nutriture. In agreement with these researchers, we found that individuals in the highest age decile, 80–89 years, as compared to those in the lowest, 60–69 years, had more than twice the prevalence of peripheral neuropathy (determined by examination).

Disability may also be modified by the presence of B12 deficiency. Though several researchers have explored disability as a function of aging [5,14,22,23,24], little research is available describing the domains of disability in relation to B12 status. In the present study, individuals with the MMA/B12 deficiency (definition 3) had 60% increased odds of disability. Matteini *et al.* [24] evaluated the association of B-vitamin markers and frailty syndrome as assessed by weight status, grip strength, endurance, physical activity, and walking speed. These researchers found that B12 insufficiency defined by MMA > 0.27 μmol/L was associated with 72% greater odds of frailty (95% CI: 0.92–3.32). Frail individuals were also significantly more likely to have decreased cognitive performance as measured by the Mini-Mental State Examination. In the present report, we found that LSA of all the disability domains was one that was most strongly associated with B12 nutriture when using the B12/MMA definition. This domain has also been strongly associated with risk of developing Alzheimer’s disease [23,25]. In a sample of 516 adults in the Chicago Health and Aging cohort, we found that elevated MMA and low B12 were predictive of faster rates of cognitive decline [26].

### Strength and Limitations

The findings of this study (presence of peripheral neuropathy, cognitive impairment, and greater disability) amongst older adults defined as B12 deficient is important because they are based on a population-based representative sample of 3105 older adults of diverse race-ethnic backgrounds in the US. Several of the signs and symptoms measured in this study increase or decrease with the aging process. However, care was taken to adjust for potential confounding factors to ensure we were measuring the effect of B12 deficiency and not of other factors. Adjustments were made for age, sex, race/ethnicity, smoking status, serum creatinine, serum ferritin, diabetes, and peripheral artery disease. Further adjustments for supplement use, coronary heart disease, and alcohol use resulted in no material differences in reported associations. Peripheral neuropathy and sensory disturbances are prevalent among those with diabetes [14], but poor vitamin B12 nutriture may also co-exist [27].

The present research is limited because older NHANES data sets, 1999–2002, were used in order to include many historically recognized functional indicators of B12 deficiency. Moreover, many of the historically recognized signs and symptoms were not available in NHANES 1999–2002, such as sensory measurement on hands (monofilament testing was only available for both feet in NHANES 1999–2001), reflexes, and autonomic assessments. Had other indicators such as ankle tendon reflex or vibratory sense or nerve conduction velocity testing been included, it might have been possible to detect more marked effects (as well as a more complete assessment of peripheral neuropathy) when the definitions of B12 deficiency were applied. As mentioned, impaired balance was one of the historically recognized signs associated with B12 deficiency. Balance was evaluated only in subjects to the age of 69 in resulting in a smaller sample in NHANES 1999–2000. In the present study we only used measurements for the most difficult condition (condition 4), which measures vestibular strengths, after vision and proprioception tests were passed. Perhaps in the selection of the most rigorous condition we missed detection of subtle proprioceptive impairments. Because of this possible limitation, no significant relationships were found for balance with any of the B12 deficiency definitions.

Finally, these analyses are based on data from a cross-sectional survey. While these observations are important on a public health level, our findings cannot be construed to reflect a cause and effect relationship. Cohort studies can provide a useful means to extend these findings by providing a temporal picture of B12 nutriture and subsequent presence or absence of symptoms or disability, such as the recent report by Leshear and coworkers [5].

## 5. Conclusions and Implications

In the present study, we have described the prevalence of vitamin B12 deficiency when described in three ways and report increased odds of several signs and disability with at least two of these definitions—definitions that include concentrations of functional biomarkers, MMA or homocysteine. Others have noted that signs and symptoms associated with B12 deficiency in a US case series nearly two decades ago and in individuals with serum B12 concentrations higher than the commonly used cutoff of <148 pmol/L [11,20,21]. The present report provides a renewed and more recent rationale for clinicians to use definitions of B12 deficiency that include homocysteine or MMA. By identifying and treating a B12 deficiency earlier, it may be possible to prevent some of the functional disabilities common to individuals as they age but possibly responsive to improved nutrition.

Because of prior evidence of the benefit of early detection, we recommend that cognitive function, peripheral neuropathy, as well as ankle tendon reflex, vibratory sense, and autonomic function be assessed in another cohort with a more complete assessment of B12 nutriture. Early detection of B12 deficiency is critical in the pursuit of improved quality of life and healthcare cost savings. These findings clearly warrants further confirmation in a setting designed to address these relationships more thoroughly. The public health consequences in a growing older population could be immense.
